# Contemporary Review of Submandibular Gland Sialolithiasis and Surgical Management Options

**DOI:** 10.7759/cureus.28147

**Published:** 2022-08-18

**Authors:** Ido Badash, Jonathan Raskin, Michelle Pei, Liuba Soldatova, Christopher Rassekh

**Affiliations:** 1 Tina and Rick Caruso Department of Otolaryngology-Head and Neck Surgery, Keck School of Medicine, University of Southern California, Los Angeles, USA; 2 School of Medicine, Oakland University William Beaumont School of Medicine, Detroit, USA; 3 Department of Otolaryngology-Head and Neck Surgery, University of Miami Miller School of Medicine, Miami, USA; 4 Department of Otolaryngology-Head and Neck Surgery, The Ohio State University Wexner Medical Center, Columbus, USA; 5 Department of Otorhinolaryngology-Head and Neck Surgery, University of Pennsylvania Health System, Philadelphia, USA

**Keywords:** operative management, sialolithiasis, salivary gland, maxillofacial surgery, submandibular gland

## Abstract

One of the most common disorders of the salivary glands is obstructive sialolithiasis. Salivary gland obstruction is important to address, as it can significantly impact patient quality of life and can progress to extensive cellulitis and abscess formation if left untreated. For small and accessible stones, conservative therapies often produce satisfactory outcomes. Operative management should be considered when stones are inaccessible or larger in size, and options include sialendoscopy, laser lithotripsy, extracorporeal shockwave lithotripsy, transoral surgery, and submandibular gland adenectomy. Robotic approaches are also becoming increasingly used for submandibular stone management. The purpose of this review is to summarize the modern-day management of submandibular gland obstructive sialolithiasis with an emphasis on operative treatment modalities. A total of 77 articles were reviewed from PubMed and Embase databases, specifically looking at the pathophysiology, clinical presentation, diagnosis, and management of submandibular sialolithiasis.

## Introduction and background

Introduction

Sialolithiasis is the most common disorder of the major salivary glands, accounting for 50% of major salivary gland diseases [1,2]. While sialolithiasis has been noted in approximately 1% of autopsy reports, clinically significant sialolithiasis is less common, with studies showing an incidence of 30 to 60 symptomatic cases requiring treatment per million individuals per year [3-5]. Patients with obstructive sialadenitis present with a history of recurrent painful periprandial swelling of the involved gland, best known as the “meal-time syndrome,” which may often be complicated by recurrent bacterial infections with fever and purulent discharge at the papilla [6]. If left untreated, salivary gland obstruction can progress to extensive cellulitis, abscess formation, and airway compromise [7].

Sialolithiasis occurs in the submandibular gland in about 80-90% of cases [8]. Historically, submandibular sialoadenectomy was the treatment of choice for complicated or recalcitrant sialolithiasis. Recent advances in minimally invasive treatment options for submandibular sialolithiasis have led to successful stone removal with high rates of gland preservation. This article provides an update on contemporary management of submandibular gland sialolithiasis, with an emphasis on minimally invasive treatment modalities including sialendoscopy and transoral robotic surgery (TORS).

Methods

We searched PubMed and Embase databases for articles published in the English language. Keywords used included “submandibular gland,” “sialolithiasis,” “salivary stone,” “sialography,” “sialendoscopy,” “lithotripsy,” “sialolithotomy,” “sialoadenectomy,” “transoral surgery,” and “transoral robotic surgery.” Original and review articles related to the pathophysiology, clinical presentation, diagnosis, and management of submandibular sialolithiasis were reviewed. A total of 77 articles were reviewed and included, with levels of evidence ranging from 1 to 5 ( Table 1).

**Table 1:** Quality rating scheme for studies and other evidence

Level of Evidence	Study Design
1	Properly powered and conducted randomized clinical trial; systematic review with meta-analysis
2	Well-designed controlled trial without randomization; prospective comparative cohort trial
3	Case-control studies; retrospective cohort study
4	Case series with or without intervention; cross-sectional study
5	Opinion of respected authorities; case reports

Pathophysiology

Sialoliths are composed of both organic substances, including glycoproteins, mucopolysaccharides, and cellular debris, as well as inorganic substances, which consist mainly of calcium carbonates and calcium phosphates [9]. Other inorganic components of sialoliths include minerals such as calcium, magnesium, phosphate, manganese, iron, and copper. The organic substances often predominate in the center of the stone, while the periphery is mostly inorganic [9].

While the pathogenesis of sialolithiasis is unknown, two major hypotheses have been proposed. Some speculate that intracellular microcalculi excreted into the canal may become a nidus for further calcification [10,11]. A second hypothesis suggests that food, debris, or bacteria in the oral cavity can migrate into the salivary ducts and become the nidus for further calcification [12]. Both hypotheses maintain that an initial organic nidus progressively grows by the deposition of layers upon layers of inorganic and organic substances [9].

The etiologic factors responsible for an increased incidence of sialolithiasis in certain individuals remain unknown. While it was traditionally believed that high calcium intake contributes to an increased risk of salivary stone formation, a study investigating the geographic distribution of water hardness showed no link between higher-mineral-content water and increased incidence of salivary stones [13]. Results of a national study in Sweden suggested that genetics may play a role, as familial clustering was common in patients with sialolithiasis. Other risk factors commonly attributed to salivary gland stone formation include dehydration and smoking, use of diuretics and anticholinergic medications, history of gout or trauma, and chronic periodontal disease [7,14,15].

Salivary stones occur predominantly in the submandibular gland, likely because Wharton’s duct has a longer course, upward salivary flow, and increased alkalinity and viscosity of the saliva when compared with Stensen’s duct [16]. The mean size of submandibular stones is 7.3 mm with an average growth rate of around 1 mm per year, although giant sialoliths measuring up to 70 mm have been described [9,17-20]. In general, stones greater than 15 mm in size are considered giant salivary gland calculi [20]. The majority of submandibular stones are located in the distal third of the duct or at the hilum of the gland, while pure intraparenchymal stones are infrequent [2]. The location of submandibular stones is important for guiding management, and a variety of diagnostic modalities may be used for submandibular stone localization.

Clinical presentation

Sialolithiasis is primarily a clinical diagnosis based on patient history and physical examination. Patients commonly report a sudden onset of swelling and pain in the affected gland that is associated with eating, referred to as “meal-time syndrome” Acute sialadenitis in the setting of sialolithiasis presents with pain, swelling, and erythema around the gland. Fevers and chills may also be present [15]. 

On physical exam, palpation of the floor of the mouth in a posterior-to-anterior direction may allow the sialolith to be seen at the opening of Wharton’s duct or palpated along its course [15]. The gland itself may be tender to palpation, particularly in the presence of sialadenitis. Additionally, compression of a salivary gland should cause clear saliva to flow from the associated duct; if this does not occur, a stone may be obstructing salivary flow. Finally, purulent discharge at the orifice raises concern for acute bacterial sialadenitis [7].

## Review

Assessment and diagnosis

Aside from patient history and palpation of the duct, various imaging techniques are available for the diagnosis of sialolithiasis (Table 2) [21]. Imaging can identify a sialolith, an abscess, or mimickers of sialolithiasis such as neoplasms. Imaging modalities include conventional sialography, computed tomography (CT), ultrasound (US), and magnetic resonance (MR) sialography [8,9]. 

**Table 2:** Imaging modalities for the diagnosis of sialolithiasis

	Conventional Sialography	Non-Contrast Computed Tomography (CT)	Ultrasound (US)	Magnetic Resonance Sialography
Advantages	Clear visualization of ductal anatomy	Commonly available High resolution Can be performed without contrast-use Ability to detect smaller stones	Commonly available Low-cost Non-invasive No radiation exposure or contrast use Able to visualize ductal dilation	Non-invasive No radiation exposure or contrast use Precise evaluation of salivary duct anatomy Ability to detect very small stones Allows concomitant evaluation of salivary gland parenchyma and surrounding soft tissues
Disadvantages	Rarely used Radiation exposure Iodine contrast use Invasive; risk of calculi dislodgement, ductal perforation, inflammation, bleeding Quality of study is highly technician-dependent Contraindicated in patients with acute sialadenitis or contrast allergy	Lower resolution for visualizing duct dilation, intraductal or glandular pathology than other modalities	Less useful for <2mm stones Low sensitivity for detecting salivary neoplasms, strictures, or other complications Quality of study is technician-dependent	Visualization of sialolith is indirect, can lead to false negatives Less sensitive for stones that do not cause full ductal occlusion Dental amalgams may limit usefulness of study
Sensitivity	Traditional 64-100% With subtraction 88-100%	98%	59-94%	91%
Specificity	Traditional 96-100% With subtraction 88-91%	88%	87-100%	94-97%
Comments		Non-contrast CT and US are often used in conjunction, as advantages of one makes up for the shortcomings of the other		

Conventional Sialography

In conventional sialography, the duct is cannulated and a radiopaque dye is injected before plain films are taken [22]. Although rarely performed today, it is still regarded as one of the best diagnostic techniques for visualizing the detailed anatomy of the salivary ducts, as it can demonstrate the main duct as well as all its branches, from primary to quaternary ones [23]. The sensitivity of conventional sialography in sialolith detection ranges from 64 to 100%, while its specificity ranges from 88 to 100%. With the use of subtraction, the sensitivity of stone detection increases to 96-100% while specificity is as high as 88-91% [24]. 

The disadvantages of sialography include exposure to ionizing radiation and iodine contrast media, pain during contrast medium insertion into the salivary ducts, and calculi dislodgement towards the gland. Additionally, the quality of the resulting image depends on the experience of the operator performing the cannulation and sialography evaluation [23]. The technique is also associated with several complications, including salivary duct perforations, inflammation, adverse reactions to iodine contrast medium, and bleeding [23]. It is also contraindicated in patients with acute sialadenitis or contrast allergy [7]. Due to these drawbacks, sialography has been largely supplanted by newer imaging modalities [23].

Computed Tomography 

High-resolution neck CT is one of the most commonly used modalities for the evaluation of salivary stones [25]. Most stones contain enough calcium to be visible with non-contrast CT, and fine cuts should be used so that small stones are not missed. Non-contrast CT has high sensitivity and specificity for salivary stone detection: a retrospective cohort study reported a sensitivity of 98% and a specificity of 88% using sialendoscopy as the reference standard [4]. It is particularly useful in cases where few small calculi are suspected that may be missed with other diagnostic modalities, especially ultrasound (US).

Two main drawbacks of non-contrast CT imaging are that it exposes the patient to ionizing radiation and provides less detail of ductal dilation and other intraductal or glandular pathologies than conventional or MR sialography [23]. Contrast-enhanced CT imaging may be performed in addition to non-contrast CT studies to provide further detail for the evaluation of complicated stone disease. However, this results in doubling radiation exposure for the patient and carries additional risks associated with intravenous contrast use such as anaphylaxis and acute kidney injury [25]. Traditionally, it has been thought that contrast-enhanced CT should not be used as a stand-alone study because of the concern that blood vessels may resemble small sialoliths and lead to false-positive diagnoses. However, a recent study by Purcell et al. reported a 98% accuracy rate for contrast-enhanced CT for the diagnosis and exclusion of salivary calculi, with the conclusion that there may be no difference in the diagnostic accuracy between contrast-enhanced and non-contrast CT studies [25]. 

Ultrasound

Ultrasound is another frequently used modality for the diagnosis of submandibular sialolithiasis. Sialoliths typically appear as echogenic round or oval structures producing an acoustic shadow on US [23]. Stones may also lead to proximal distension of the duct, which may be seen in US. The detection of fine stones may be helped by sialogogue injection, which causes salivary duct dilatation and thus facilitates sialolith visualization [23]. 

US is most suited for stones larger than 2 mm, greater than 90% of which can be detected by ultrasound [26]; stones smaller than 2 mm may not produce any acoustic shadow and are therefore difficult to detect [23]. US has been reported to have a sensitivity ranging from 59% to 94% and a specificity ranging from 87% to 100% for the detection of submandibular sialolithiasis [4,23,24,27]. The differential diagnoses that can arise when using US for sialolithiasis include sarcoidosis, Sjögren syndrome, disseminated lymphoma, and hematogenous metastasis [27]. Advantages of US include its noninvasive nature, relatively low cost, and lack of radiation exposure. Disadvantages include the need for an experienced operator and low sensitivity for detecting salivary gland neoplasms or stone-related complications such as strictures [7]. 

US and non-contrast CT can also be used in combination for the detection of submandibular sialoliths, which allows for the advantages of one modality to compensate for the disadvantages of the other [4]. CT imaging is more sensitive for individual sialoliths and can illustrate multiple stones, whereas US can demonstrate duct dilation when stones are difficult to visualize directly. CT also provides complementary information regarding possible glandular abscess or tumor, while obtaining US provides radiologists a point of comparison should they be called into the operating room to assess for stones [4]. 

Magnetic Resonance (MR) Sialography

MR sialography is a noninvasive alternative to conventional sialography in that it does not require salivary duct cannulation or ionizing radiation and contrast exposure. Unlike conventional sialography, it may also be carried out during acute inflammation of the salivary gland [23]. Studies of MR sialography suggest that it may have superior sensitivity to US and a lower procedural failure rate than conventional sialography [24,28]. The sensitivity and specificity of MR imaging sialography are reported to be 91% and 94%-97% respectively [29].

The main advantages of MR sialography are the precise evaluation of the salivary duct including proximal branches, and the detection of very small stones which may not be found with other diagnostic techniques [30]. Additionally, it does not require an experienced operator and permits concomitant evaluation of the salivary glandular parenchyma [31]. The primary drawback of MR sialography is that the diagnosis of sialolithiasis is almost entirely indirect, relying on findings such as ductal obstruction with signal loss and prestenotic dilation. Therefore, some small stones that do not cause full occlusion, such as those found near duct openings or in intraglandular ducts, may remain undetected. Dental amalgams may also lead to distortion artifacts with this modality [28,30]. 

Treatment

Primary Care Management

Conservative management is the mainstay of treatment in the majority of patients presenting to a primary care clinician for sialolithiasis [32]. Patients should be instructed to maintain hydration, apply heat to the involved area, use nonsteroidal anti-inflammatory drugs to reduce pain and inflammation, and massage the gland to promote ductal outflow. Nonpharmacologic agents that promote salivary flow, such as lemon wedges and tart candies, may be helpful. After the resolution of the episode, risk factors for sialolithiasis should be identified and modified to prevent future episodes [7]. 

If sialadenitis is suspected because of increasing pain, fever, or purulent drainage, anti-staphylococcal antibiotics should be administered [15,33]. If there is not an improvement in symptoms within a week of treatment, a culture of any ductal discharge should be obtained and the antibiotic coverage should be broadened until culture results are available [15,34]. US or CT imaging with contrast may be performed if there are signs suggestive of an abscess, such as fluctuance, erythema, and warmth [7].

Interventional Management

Patients who have symptoms of obstruction lasting more than a few days should be considered for operative management, as should those with recurrent episodes of sialolithiasis due to risk for chronic sialadenitis and loss of glandular function [7]. Patients with sialadenitis that worsens or shows no improvement with antibiotics also require operative evaluation as they are at risk for the development of salivary gland abscess and spread of the infection to the floor of the mouth, potentially leading to airway compromise [35].

The objective of interventions for submandibular stone extraction is generally to save the gland. The classic algorithm first reported by Marchal et al. in 2003 is still commonly used, which recommends sialendoscopy with wire basket extraction for stones smaller than 4 mm, and laser lithotripsy with sialendoscopic extraction for stones greater than 4 mm.

**Figure 1:**
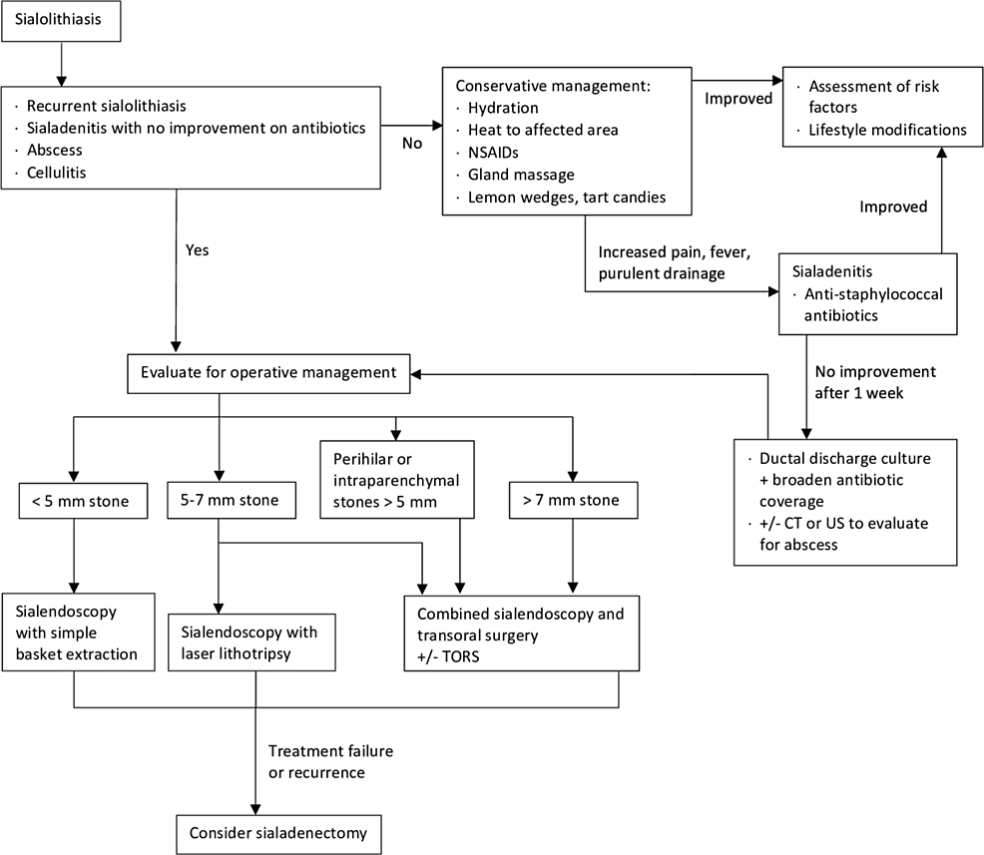
An updated treatment algorithm for the management of submandibular sialolithiasis CT: Computed Tomography; US: Ultrasound; TORS: Transoral Robotic Surgery

With the advent of new surgical approaches, refinement of existing interventions leading to decreased associated morbidities, and additional studies reporting on the efficacy of the various treatment options, an updated treatment algorithm is necessary for the management of submandibular sialolithiasis (Figure 1). We recommend that stones up to 5 mm can be removed with sialendoscopy using simple basket extraction. Stones between 5 and 7 mm should be removed either with combined sialendoscopy and transoral surgery or using laser lithotripsy with sialendoscopy. Stones larger than 7 mm and those near the hilum or partially inside the gland are amenable to combined approaches. Transoral robotic surgery can also be used to facilitate combined approaches. The following sections discuss these treatment modalities in detail.

Sialendoscopy

Sialendoscopy is a minimally invasive technique for submandibular stone visualization and removal that has the potential to avoid nerve injury, facial scarring, and oral injury associated with traditional open surgery (Table 3) [36]. The sialendoscope combines a delicate semi-rigid fiberoptic endoscope, an irrigation port, and a working channel in a single instrument. The endoscope broadcasts high-definition images to a monitor. Irrigation is used to dilate the ducts, permitting exploration of the branches of the salivary system. The working channel is a conduit for instruments, including custom-designed baskets, graspers, and guidewires that can be used to remove salivary stones [9].

**Table 3:** Minimally invasive treatment options for sialolithiasis ESWL: Extracorporeal Shockwave Lithotripsy; FDA: Food and Drug Administration

	Sialendoscopy	Sialendoscopy with laser lithotripsy	Extracorporeal shockwave lithotripsy (ESWL)
Best uses	<5mm stones Stones that are free-floating within duct	5-7mm stones	Any sized stone, although most effective for <7mm stones
Advantages	Minimally invasive Direct, high-definition intra-ductal visualization Variety of extraction instruments available Stones can be extracted intact	Direct, high-definition intra-ductal visualization Ability to remove larger sized stones while avoiding more invasive surgical operations	Easy to perform, in-office procedure Repeatable Very well-tolerated Stone fragments may pass spontaneously, thereby avoiding operating room and anesthesia
Disadvantages		Injuries to surrounding soft tissues	Possibility of incomplete stone clearance leading to recurrence Injuries to surrounding soft tissues
Success rate	>80%	81-100%	26-81%
Complication rate	3%	13%	N/A
Possible complications	Ductal strictures Perforations Ranula formation Lingual, facial nerve injuries Infection Bleeding	Ductal strictures Perforations Thermal injury to nerves and vessels (may be avoided with continuous cold saline rinsing)	
Comments		Thermal injuries may be minimized with continuous saline rinsing and avoiding the duct wall with the laser Ductal stenosis can be prevented with stent placement	Sialendoscopy often performed following ESWL for complete stone fragment removal Not FDA approved in United States

The advent of sialendoscopy has significantly reduced the number of salivary glands removed due to sialolithiasis [2,37,38]. Submandibular sialoliths of up to 5 mm in diameter can be successfully removed through sialendoscopy alone, and the technique is especially useful for mobile stones lying freely in the duct lumen. When used for these indications, submandibular stones can be extracted under endoscopic control without additional interventions or fragmentation in greater than 80% of cases [37,39-42]. 

Sialoliths may be mechanically removed by a basket, mini forceps, grasper, or balloon [43,44]. Several factors are involved in choosing an instrument, including mobility, connection to the ductal wall, size, and ability to bypass stones [43]. For freely floating stones, endoscopic removal is most commonly performed with the use of a basket. Balloons also are suitable tools for the removal of small mobile sialoliths. In cases in which the sialolith cannot be bypassed, mini forceps or a grasper can be used to remove the stone [45].

While considered a safe procedure, complications have been reported following sialendoscopy. The overall complication rate has been reported to be around 3%, which includes ductal strictures, perforations, ranula formation, lingual and facial nerve injuries, infection, and bleeding [43,46]. Many of these complications, as well as treatment failure, can be avoided by selecting patients most amenable to sialendoscopy alone, which are primarily those with stones <5 mm in size. 

Sialendoscopy With Laser Lithotripsy 

Submandibular stones between 5-7 mm in size may be fragmented in the duct lumen using endoscopically-guided laser lithotripsy before manual extraction [47]. Holmium:YAG (yttrium-aluminum-garnet) laser-assisted lithotripsy is the most common variation of this technique utilized for salivary gland stones and has shown to be an effective, safe, and relatively simple method for treating larger submandibular sialoliths [48]. Results from recent studies show that the rate of successful stone extraction for carefully selected patients undergoing sialendoscopy and laser lithotripsy ranges from 81% to 100% [49-52]. 

One of the major advantages of this method is the direct visualization of the stone as well as assessment of the ductal system before, during, and after the intervention, as compared to extracorporeal shockwave lithotripsy (ESWL) [48]. By using laser lithotripsy with sialendoscopy, larger stones that otherwise would not be amenable to sialendoscopy alone may be removed. This also obviates the need for more invasive surgical management. However, there are still risks of perforation, stricture, and thermal injuries to nerves and vessels with this technique, which may occur at rates as high as 13% [53]. With continuous cold saline rinsing and avoidance of exposing the ductal walls to the laser, these risks can be minimized [37]. Stents may also be placed after laser utilization to prevent the formation of ductal stenosis [40].

Extracorporeal Shockwave Lithotripsy (ESWL)

Another option for the fragmentation of large sialoliths is to perform ESWL. For this technique, US imaging is used to focus an electromagnetic or piezoelectric shock wave on a submandibular sialolith to fragment the stone. US is also used to continuously monitor the degree of stone fragmentation during each therapeutic session and to avoid lesions to the surrounding tissues [47]. ESWL permits fragmentation of stones of any size and location, which are then excreted either spontaneously by salivary flow through Wharton’s duct or manually with sialendoscopy.

Since ESWL is performed as an in-office procedure, it offers several advantages over other interventions in that it is easy to perform, repeatable, and well-tolerated. Most notably, if stone fragments can pass spontaneously, the greatest benefit of ESWL is the avoidance of anesthesia in the operating room. The main drawback of ESWL is that stones often cannot be completely cleared by salivary flow and residual fragments can cause recurrences. For this reason, sialendoscopy is often performed following ESWL treatment, although this combination precludes the advantages of using ESWL as an in-office procedure [47]. 

The effectiveness of ESWL for complete stone clearance ranges from 26-81% [47,54-56]. According to a large study of over 400 patients by Capaccio et al., complete clearance of residual stone fragments was achieved in 28% of cases with a distal location and 49% of cases with a hilo-parenchymal stone location [47,54]. In general, the success rate for ESWL drops with an increasing stone diameter and perihilar or intraparenchymal submandibular gland stones of < 7 mm are the best candidates for ESWL in countries where it is approved [37,47]. Currently, the technique is not approved by the Food and Drug Administration for use in the United States.

Combined Approach of Open Transoral Surgery With Sialendoscopy

**Figure 2:**
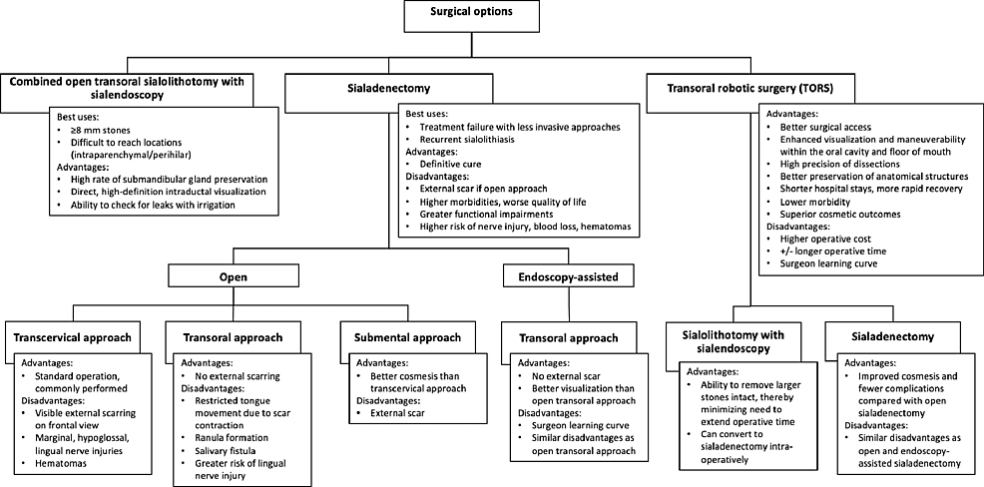
Surgical treatment algorithm for the management of submandibular sialolithiasis References: Bodner L [8]; Zenk et al. [21]; Capaccio et al. [59]

Larger (≥8 mm) submandibular stones as well as those that are difficult to access with sialendoscopy alone, such as intraparenchymal and perihilar stones, can be removed using a combined approach pairing sialendoscopy and transoral stone removal (Figure 2) [57,58]. First described by Francis Marchal in 2007, this technique involves the use of the sialendoscope to localize the sialolith before performing transoral surgery for stone extraction [42]. Sialendoscopy can again be performed after stone removal to check for additional sialoliths and to remove stone remnants. Successful stone removal rates of 69%-100% have been reported using the combined approach, with a recent large study of the combined approach for hilar or parenchymal submandibular stones by Capaccio et al. reporting a stone removal rate of 98.5% [59]. Furthermore, submandibular gland preservation rates as high as 95% using the combined approach have been published [18,42,58-60]. 

While large submandibular sialolith removal can be attempted with transoral surgery alone, this can be challenging when stones are in a hilo-parenchymal location, making stone localization difficult. Additionally, limited exposure to the floor of the mouth due to reduced mouth opening, large teeth, or obesity will also make stone removal challenging without sialendoscopy. This further complicates the identification and preservation of the lingual nerve and the placement of sutures in the salivary duct for repair after sialolithotomy [57,58].

Therefore, the combined approach technique offers several advantages over transoral stone removal alone. First, stones can be visualized directly with the sialendoscope and do not rely solely on bimanual palpation. Second, fixation of the stone with the basket and manipulation to a better location within the duct can facilitate precise surgical removal. Additionally, the ability to inspect for stone fragments after sialolith extraction helps detect incomplete treatment and prevent sialolith recurrence. Finally, the ability to irrigate the site of duct repair to check for leaks is another advantage of combining sialendoscopy with transoral removal [58]. As a result of these additional benefits, the combined approach is now the standard of care for larger submandibular stones ≥8 mm in size.

Sialadenectomy

Only 2% to 5% of patients with submandibular sialolithiasis require submandibular gland excision [6,61]. Today, sialadenectomy for submandibular sialolithiasis is primarily reserved for cases where combined or minimally invasive approaches are unsuccessful. It may also be utilized in patients with recurrent stones or for patients who cannot tolerate a second procedure. On removal of the gland and duct, no further obstructive symptoms will occur, resulting in a definitive cure for unilateral sialolithiasis. 

The transcervical approach to sialadenectomy is the most common, as it provides direct exposure to the gland and can be performed relatively quickly. However, complications such as scarring, nerve injury, and hematoma may occur [62,63]. To minimize the morbidities associated with the conventional transcervical approach, other techniques for sialoadenectomy may be used. Gland resection via an intraoral approach minimizes visible scarring but has associated risks of ranula formation, salivary fistula, lingual nerve injury, and scar contracture limiting tongue movement [64,65]. Submental approaches to gland excision have also been described with possibly improved cosmetic results compared with the transcervical approach [66]. Finally, endoscope-assisted submandibular sialadenectomy through the transoral approach is another option for sialadenectomy, which further minimizes incision length, scarring, blood loss, and risk of nerve injury [63]. 

Sialoadenectomy should be avoided whenever possible for several reasons. As the gland's function is completely lost after this procedure, patients’ quality of life may be significantly impacted. While young patients may compensate for this loss with secretions from the other salivary glands, the function of these other glands in older patients may already be limited; as such, sialadenectomy can lead to xerostomia and significant functional impairments in eating and swallowing within this population [62]. Gland excision is a more difficult procedure than other minimally invasive techniques and therefore has a greater risk of injury to the lingual, marginal mandibular, and hypoglossal nerves [7]. Visible scarring is also a concern with sialadenectomy, especially when performed through the common transcervical approach [63].

Robotic Surgery for Submandibular Sialolithiasis

Transoral robotic surgery (TORS) utilizing the da Vinci surgical system has been utilized for various diseases of the head and neck, including resection of oncologic disease of the oropharynx, hypopharynx, larynx, and parapharyngeal spaces, as well as for salivary gland disorders, including removal of floor of mouth ranulas, tumors of the submandibular gland, and salivary gland stones [57,67-70]. Robotic-assisted procedures applied specifically to submandibular stone management include the combined approach of TORS and sialendoscopy, as well as robotic sialadenectomy [57]. The use of TORS is an appealing alternative to open approaches for salivary gland diseases that may offer better surgical access, less scarring with improved cosmesis, diminished blood loss, shorter hospital stays, and lower morbidity overall [57,71]. According to a study conducted by Tampino et al., in the combined TORS and sialendoscopy approach, there was a 94% success rate in their 33 patients, with 15.1% of patients experiencing transient tongue paresthesia [72].

The primary advantages of TORS over open approaches for submandibular stone management are that it overcomes the challenges of the reduced operative field between the tongue and the mandible, and helps prevent injury to the delicate structures in the floor of the mouth [57,70]. The magnification and dexterity provided by the robotic surgical system allow precise dissection and preservation of the lingual nerve, hypoglossal nerve, and Wharton’s duct. According to Cammaroto et al., the TORS approach is recommended due to its improved hemostatic control of the facial artery, which can be difficult to manage in an intraoral open approach [73].

Additionally, compared to traditional transoral surgery, TORS offers an improved and direct view of the floor of the mouth, and thus allows the entire treatment team to collaborate and participate in the surgery [57]. The ability to perform a 4-handed surgery is an added benefit that facilitates working with multiple instruments in the small field of the oral cavity [68].

Robotic-assisted sialolithotomy is often used in combination with sialendoscopy, utilizing the da Vinci robot system for the surgical portion of the procedure [57,70]. Transoral robotic sialolithotomy has the advantage of removing larger stones intact, since extracorporeal and laser lithotripsy may increase the risk of injury to surrounding soft tissues while also adding substantially to operative time if the fragments become embedded in the ducts [74-76]. If the stone cannot be removed, the TORS approach also facilitates subsequent sialadenectomy. Removal of large submandibular stones through a combined approach with TORS and sialendoscopy has been successfully performed in multiple institutions, though a 2% rate of lingual nerve damage has been reported with the combined approach when compared to the 0% of damage in the sole TORS approach [57,70,72,76].

Robotic-assisted sialadenectomy is another minimally invasive surgery with distinct benefits over open alternatives. Lee et al. described a postauricular approach for robotic sialadenectomy and reported satisfactory cosmetic results, decreased operative times compared to endoscopic gland resection, and no postoperative complications [74,75,77]. A similar study performed by Singh et al. found that while operative times were longer and had more drainage in robotic sialadenectomy compared to traditional transcervical approaches, cosmetic outcomes were significantly improved [74]. Finally, DeVirgilio et al. published a case series of patients undergoing robotic sialadenectomy with a modified postauricular facelift approach and found less scarring and improved cosmetic outcomes compared with endoscopic gland resections while describing no complications [75]. 

## Conclusions

The management of submandibular sialolithiasis has undergone enormous changes over the last several decades. With the introduction of minimally invasive techniques for stone management, including sialendoscopy and robotic-assisted surgery, even larger sialoliths can now be removed with minimal surgical morbidity and high gland preservation rates of greater than 95%. In general, stones up to 5 mm can be removed with sialendoscopy alone, stones between 5 and 7 mm are more amenable to sialendoscopy with laser lithotripsy, and stones larger than 7 mm, as well as perihilar or intraparenchymal stones, can be treated with sialendoscopy-assisted transoral surgery. Robotic surgery is also becoming increasingly popular for salivary stone management and may facilitate both sialolithotomy and sialadenectomy. If treatment is performed according to treatment algorithms incorporating these techniques, most patients can be successfully cured of sialolithiasis with minimal morbidity, complications, or recurrence.
